# The role of nitric oxide pathway in arginine transport and growth of IPEC-1 cells

**DOI:** 10.18632/oncotarget.16267

**Published:** 2017-03-16

**Authors:** Hao Xiao, Liming Zeng, Fangyuan Shao, Bo Huang, Miaomiao Wu, Bie Tan, Yulong Yin

**Affiliations:** ^1^ National Engineering Laboratory for Pollution Control and Waste Utilization in Livestock and Poultry Production, Key Laboratory of Agro-ecological Processes in Subtropical Region, Institute of Subtropical Agriculture, Chinese Academy of Sciences, Changsha, China; ^2^ University of the Chinese Academy of Sciences, Beijing, China; ^3^ Science College of Jiangxi Agricultural University, Nanchang, China; ^4^ Faculty of Health Sciences, University of Macau, Macau SAR, China; ^5^ Diagnostic Medicine/Pathobiology, College of Veterinary Medicine, Kansas State University, Manhattan, KS, USA; ^6^ Laboratory of Animal Nutrition and Human Health, School of Biology, Hunan Normal University, Changsha, China; ^7^ College of Animal Science, South China Agricultural University, Guangzhou, China

**Keywords:** arginine, nitric oxide synthase, mTOR, intestinal porcine epithelial cells (IPEC)

## Abstract

L-Arginine itself and its metabolite-nitric oxide play great roles in intestinal physiology. However, the molecular mechanism underlying nitric oxide pathway regulating L-Arginine transport and cell growth is not yet fully understood. We report that inhibition of nitric oxide synthase (NOS) significantly induced cell apoptosis (*p* < 0.05), and promoted the rate of Arginine uptake and the expressions of protein for CAT-2 and y^+^LAT-1 (*p* < 0.05), while reduced protein expression of CAT-1. And NOS inhibition markedly decreased the activation of mammalian target of rapamycin (mTOR) and PI3K-Akt pathways by Arginine in the IPEC-1 cells (*p* < 0.05). Taken together, these data suggest that inhibition of NO pathway by L-NAME induces a negative feedback increasing of Arginine uptake and CAT-2 and y^+^LAT-1 protein expression, but promotes cell apoptosis which involved inhibiting the activation of mTOR and PI3K-Akt pathways.

## INTRODUCTION

Arginine is a semi-essential amino acid that serve as an important role in diverse physiological function ranging from regulation of animal nutrition metabolism to the process of growth and development [1–5]. There is evidence showing that arginine stimulates anabolic cell pathways, such as protein synthesis, proliferation, and migration [3, 6, 7]. In rat intestinal epithelial cell, arginine stimulates downstream targets of mTOR-p70 ribosomal protein S6 (p70 S6) kinase and eukaryotic initiation factor 4E-binding protein 1 (4E-BP1) [8]. Recent studies showed that arginine stimulated the mTOR signaling pathway and protein synthesis in porcine trophectoderm cells [9] and LPS-treated enterocytes [3].

Arginine is also a substrate for endothelial nitric oxide synthase (eNOS) to produce the important vasoprotective molecule nitric oxide (NO) to regulate vital metabolic pathways [10]. NO plays great roles in regulation of arginine in growth and proliferation of intestinal porcine epithelial cell (IPEC). Arginine could stimulate cell migration via a mechanism requiring NO and phosphorylation of p70 S6 kinase [11]. Further study determined that the arginine-mediated activation of protein synthesis and mTOR pathway is independent on NO [12]. However, the effects of L-Arginine-NO pathway in the proliferation of cell, the expression of arginine transporters and the activation of mTOR signaling pathway have not been well characterized.

We hypothesized that NO pathway might play an important role in the regulation of Arginine utilization and cell growth in the intestinal porcine epithelial cells (IPEC-1). The present study was designed to test this hypothesis using L-NAME to inhibit L-Arginine-NO pathway.

## RESULTS

### L-NAME inhibits the Arginine-NO pathway

**Figure 1:**
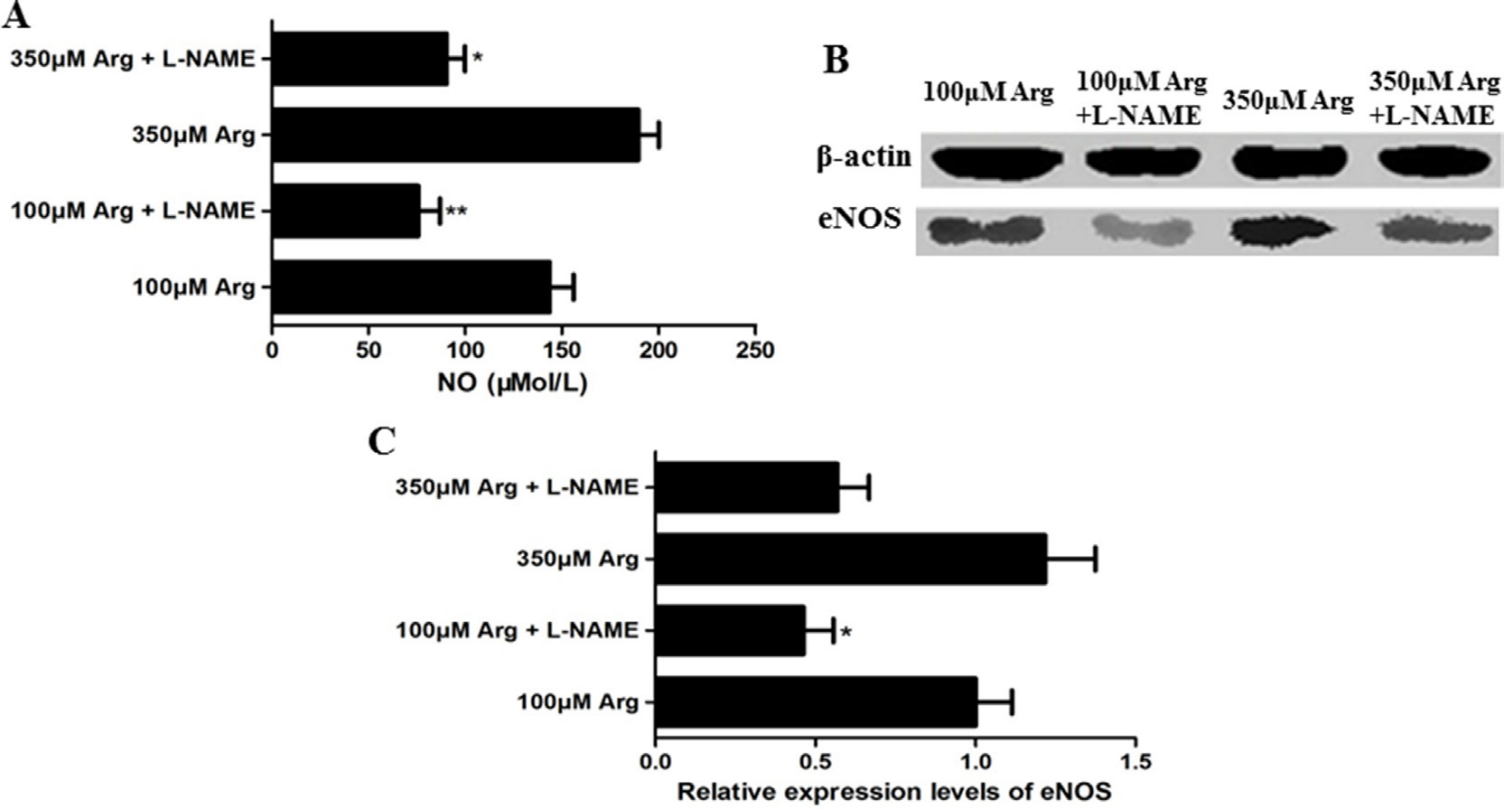
Effects of L-NAME on the Arg-NO pathway (**A**) Effects of L-NAME on the content of NO. (**B**) Expression of phosphorylated eNOS was analyzed by western blotting, with β-actin used as the internal control. (**C**) Relative protein levels for phosphorylated eNOS. Cells were treated with 100 μM Arg, 350 μM Arg, 100 μM Arg+400 μM L-NAME, 350 μM Arg+400 μM L-NAME. Data are the mean ± SEM of at least three independent experiments. *P < 0.5 and **P < 0.01.

L-NAME was used to inhibit Arginine-NO pathway, and relative proteins of eNOS and the content of NO were measured. Compared with the control (100 μM Arginine group), 350 μM Arginine medium significantly promoted (*p* < 0.05) the content of NO and the relative protein levels of total eNOS. The addition of L-NAME markedly decreased (*p* < 0.05) the content of NO and the relative protein levels of total eNOS (Figure 1).

### Inhibition of Arginine-NO pathway induces cell apoptosis

**Figure 2:**
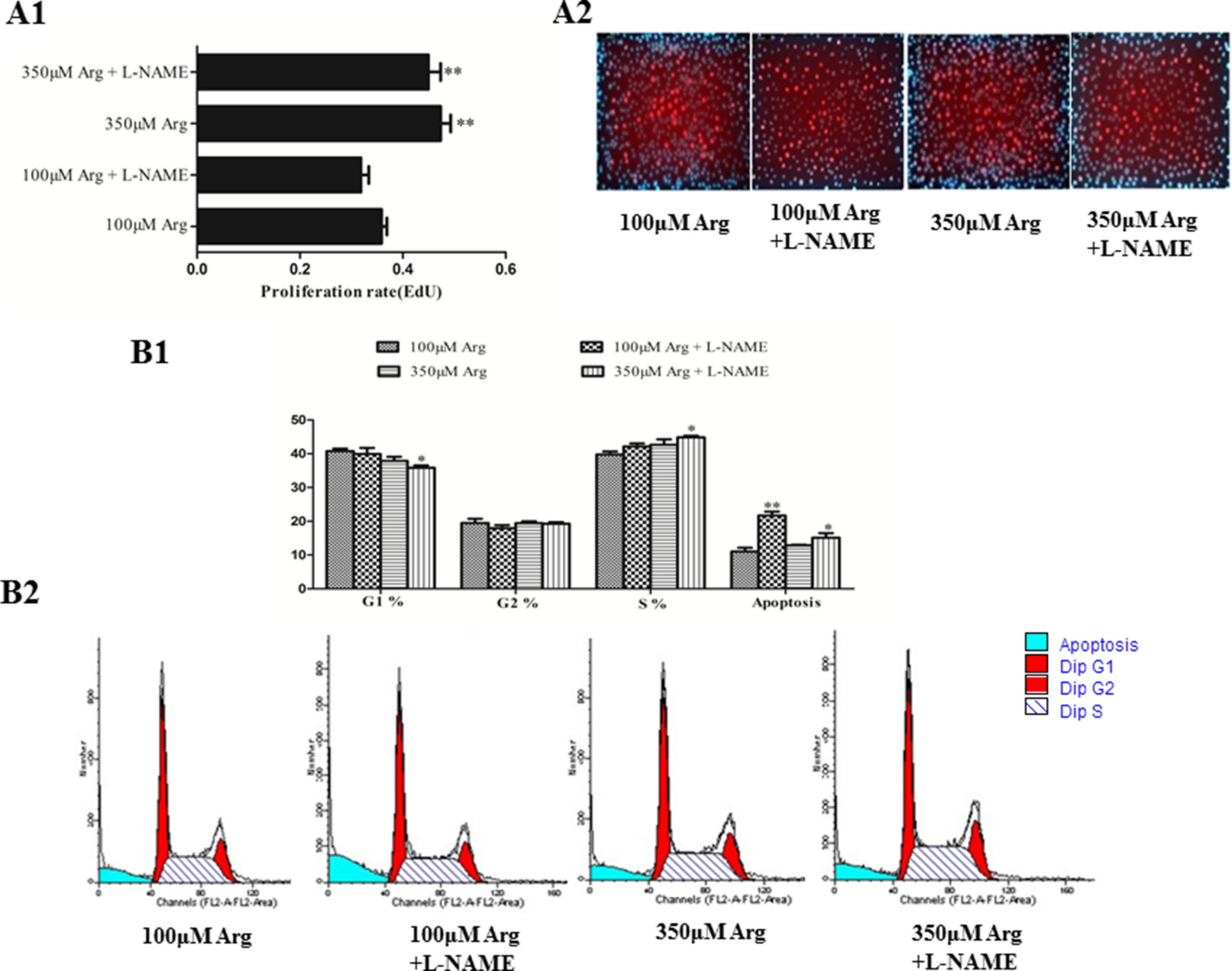
Effects of inhibition of Arg-NO pathway on cell proliferation (**A**) The EdU measurement. (**B)** Effects of inhibition of Arg-NO pathway on cell cycle distribution. Cells were treated with 100 μM Arg, 350 μM Arg, 100 μM Arg+400 μM L-NAME, 350 μM Arg+400 μM L-NAME. Data are the mean ± SEM of at least three independent experiments.*P < 0.5 and **P < 0.01

As illustrated in Figure 2, arginine at 350 μM increased (*p* < 0.05) cell proliferation compared with the control. Compared with the control, addition of L-NAME increased (*p* < 0.05) cell apoptosis, while no effect on cell proliferation, the percentage of cells in G_1_ and S phase.

### Inhibition of Arginine-NO pathway promotes rate for arginine transportation

**Figure 3:**
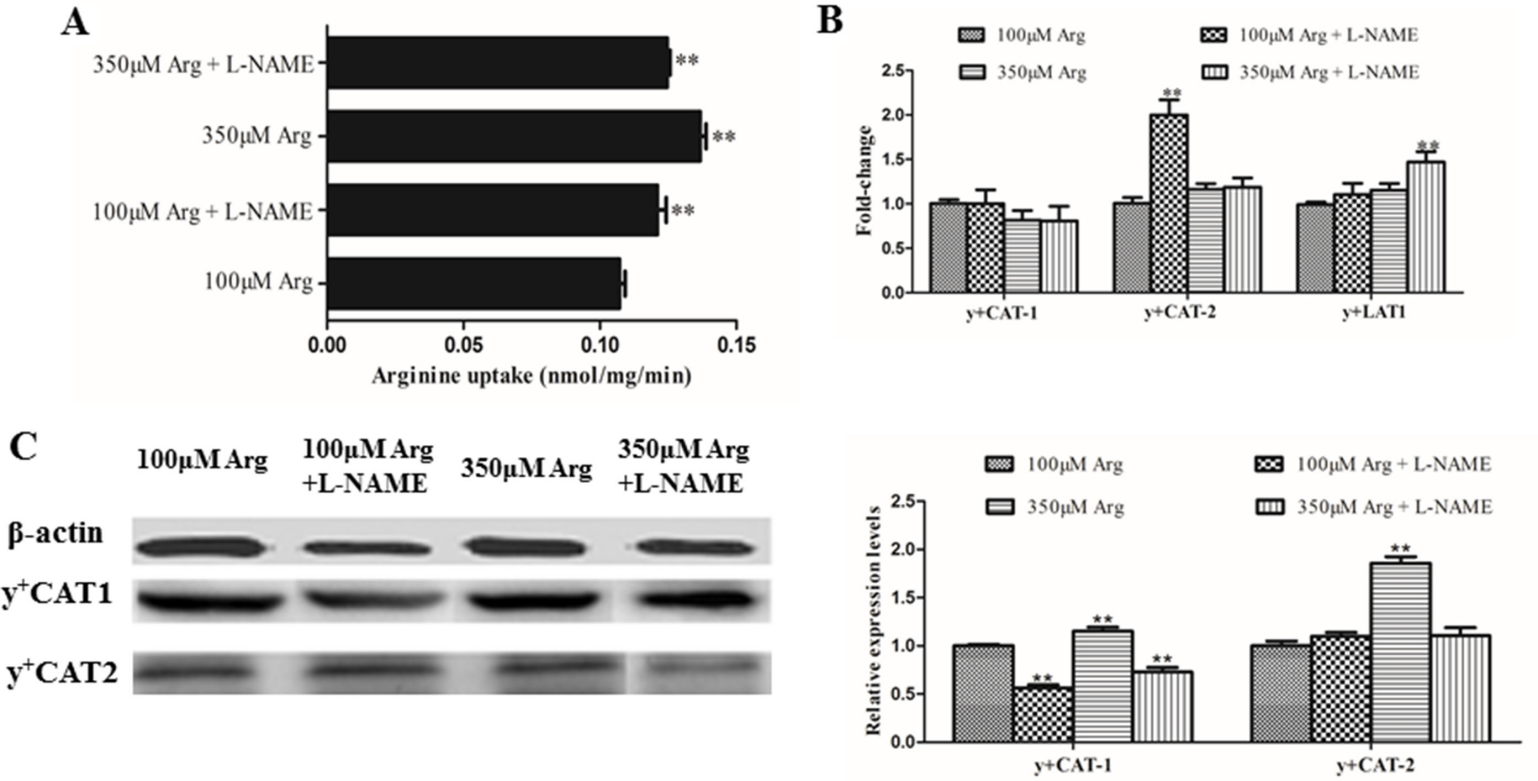
Effects of inhibition of Arg-NO pathway on arginine transport rate (**A**) Effects of inhibition of Arg-NO pathway on arginine uptake. (**B**) The mRNA levels of y+CAT-1, y+CAT-2, and y+LAT-1 were measured by RT-PCR, with β-actin used as the internal control. (**C**) Expression and relative protein levels of y+CAT-1 and y+CAT-2 was analyzed by western blotting, with β-actin used as the internal control. Cells were treated with 100 μM Arg, 350 μM Arg, 100 μM Arg+400 μM L-NAME, 350 μM Arg+400 μM L-NAME. Data are the mean ± SEM of at least three independent experiments.*P < 0.5 and **P < 0.01.

As shown in Figure 3, we found that arginine at 350 μM increased (*p* < 0.05) the arginine uptake and protein levels of CAT-1 and CAT-2 compared with the control. Addition of L-NAME significantly improved (*p* < 0.05) the arginine uptake and the mRNA level of CAT-2 and y+LAT-1, but decreased (*p* < 0.05) protein levels of CAT-1 compared with the control.

### Inhibition of Arginine-NO pathway inhibits the PI3K-Akt-Bcl2 pathway and mTOR pathway

**Figure 4:**
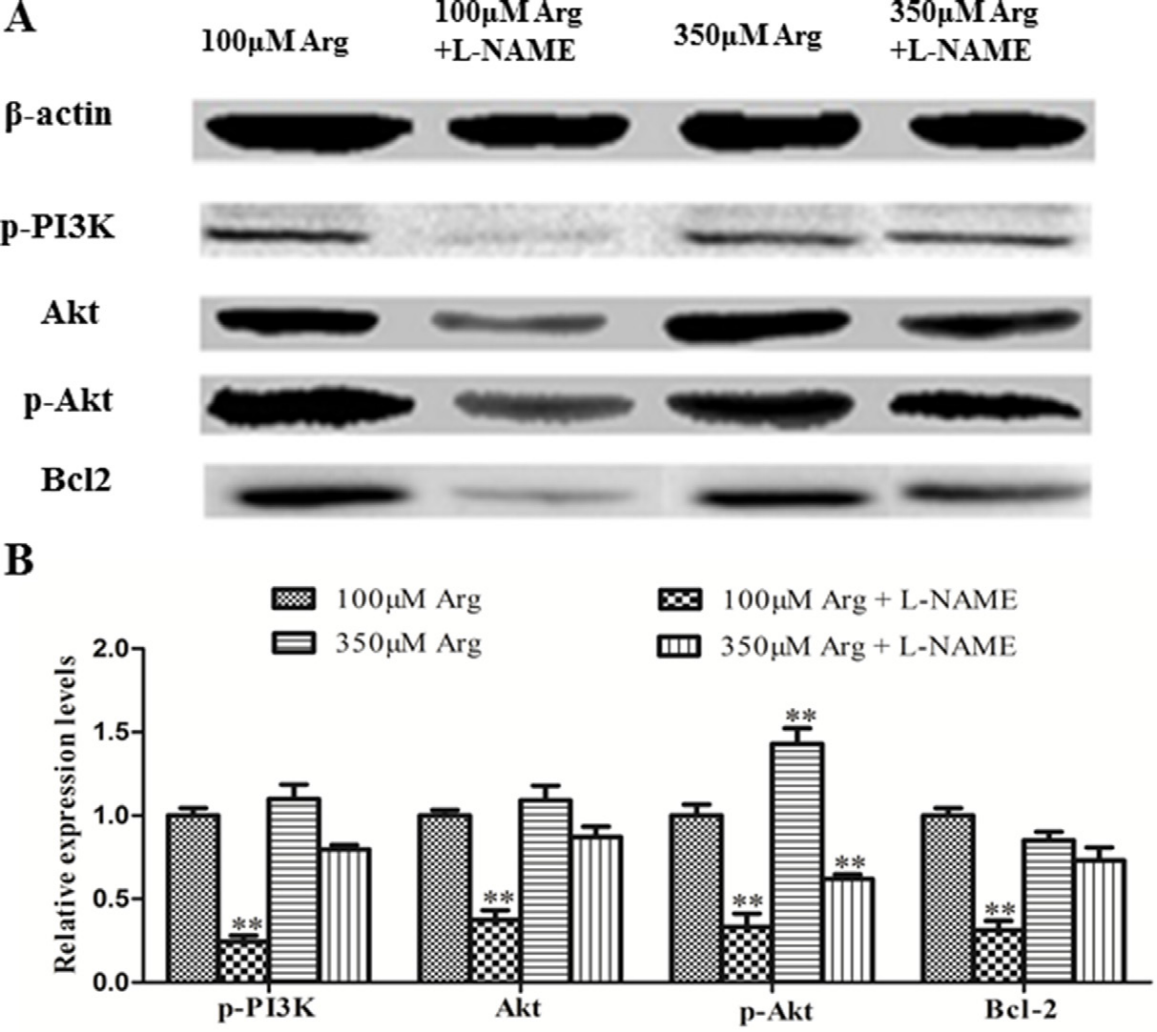
Effects of inhibition of Arg-NO pathway on PI3K-Akt-Bcl2 pathway (**A**) Expressions of phosphorylated PI3K, Akt, phosphorylated Akt and Bcl2 were analyzed by western blotting, with β-actin used as the internal control. (**B**) Relative protein levels for phosphorylated PI3K, Akt, phosphorylated Akt and Bcl2. Cells were treated with 100 μM Arg, 350 μM Arg, 100 μM Arg+400 μM L-NAME, 350 μM Arg+400 μM L-NAME. Data are the mean ± SEM of at least three independent experiments. *P < 0.5 and **P < 0.01.

**Figure 5:**
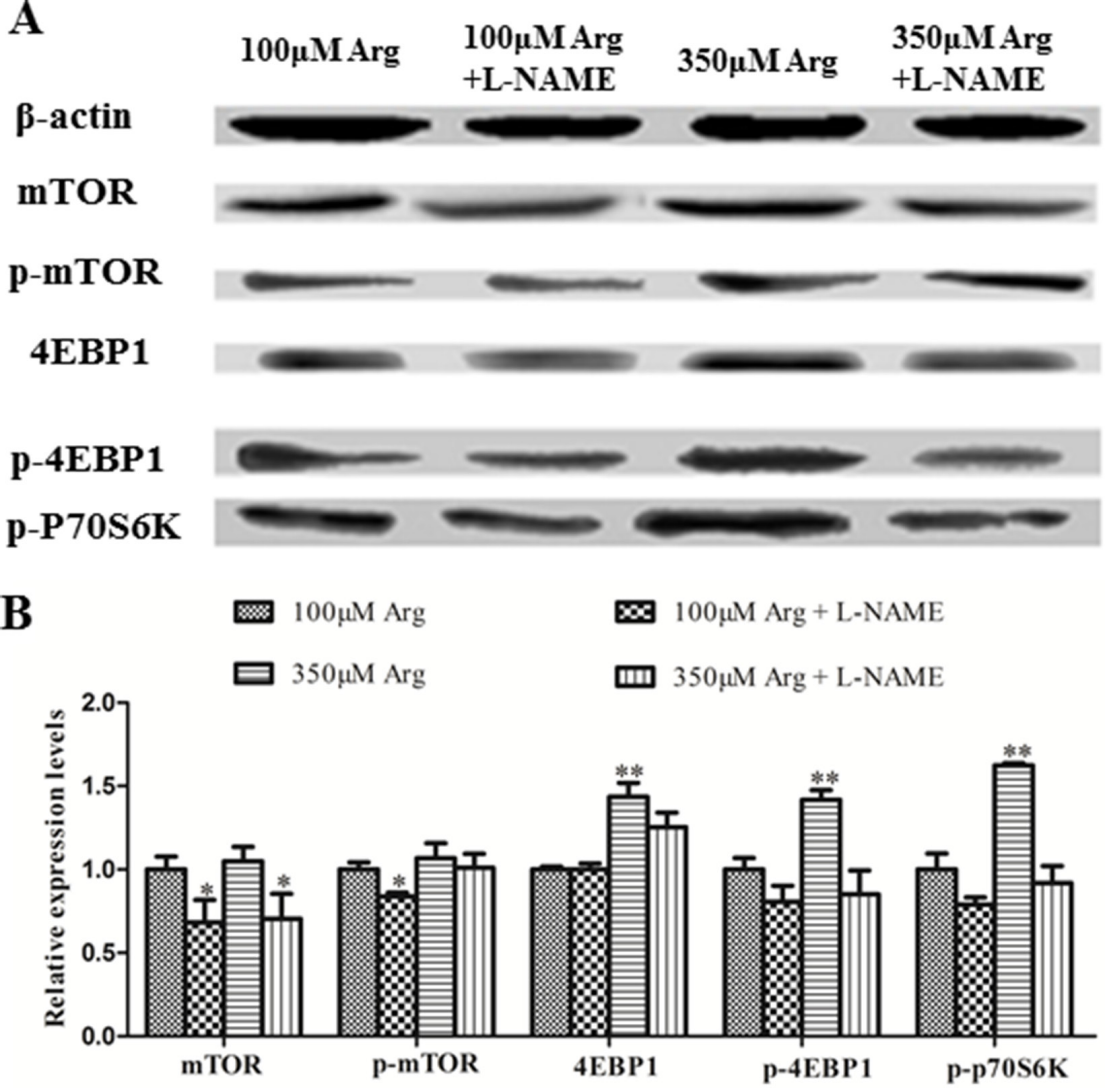
Effects of inhibition of Arg-NO pathway on mTOR pathway (**A**) Expressions of 4EBP1, phosphorylated 4EBP1, phosphorylated p70S6 Kinase, mTOR, and phosphorylated mTOR were analyzed by western blotting, with β-actin used as the internal control. (**B**) Relative protein levels for 4EBP1, phosphorylated 4EBP1, phosphorylated p70S6 Kinase, mTOR, and phosphorylated mTOR. Cells were treated with 100 μM Arg, 350 μM Arg, 100 μM Arg+400 μM L-NAME, 350 μM Arg+400 μM L-NAME. Data are the mean ± SEM of at least three independent experiments. *P < 0.5 and **P < 0.01.

Compared with the control, 350 μM Arginine medium increased (*p* < 0.05) 4EBP1, phosphorylated 4EBP1 and phosphorylated p70S6 kinase, while addition of L-NAME to 100 μM Arginine medium significantly decreased (*p* < 0.05) protein levels for mTOR, phosphorylated mTOR, phosphorylated PI3K, Akt, phosphorylated Akt and Bcl2. Addition of L-NAME to 350 μM Arginine medium decreased (*p* < 0.05) protein levels for 4EBP1, phosphorylated 4EBP1 and phosphorylated p70S6 kinase (Figure 4, 5).

## DISCUSSION

As an indispensable amino acid in almost all neonatal mammals, arginine has an important role in intestinal physiology and somatic growth [13]. Our previous study found that elevated arginine concentrations enhanced DNA synthesis, cell-cycle progression, and mitochondrial bioenergetics in LPS-treated intestinal epithelial cells through mechanisms involving activation of the PI3K-Akt pathway [14]. And Arginine supplementation could decrease expression of the proteins for NF-κB, MAPK, and PI3K-Akt signaling pathways but activated p38 and c-Jun N-terminal protein kinase in the jejunum and the ileum [15]. Lenaerts et al. reported that arginine regulated the expression of proteins of proliferation and apoptosis in Caco-2 cells [16]. Our results showed that Arginine could markedly promote the proliferative capacity of IPEC-1 cells, while addition of L-NAME has no effect on DNA synthesis and cell cycle. Moreover, phosphatidyl inositol 3-kinase (PI3K) and Akt are both well-known upstream regulators of the mTOR signaling pathway in mammalian cells [17], and the PI3K/Akt pathway provides essential signaling for cell survival and proliferation. The BCL-2 family of proteins are both pro- as well as anti-apoptotic molecules and BCL-2 is an anti-apoptotic molecule [18]. Previous study showed that long term overproduction of NO released from arginine could enhance apoptosis by activating the caspase family proteases, producing the release of mitochondrial cytochrome c [19]. According to our flow cytometry results, L-NAME may induce the cell apoptosis. Then relative protein expressions were suggested that L-NAME might induce the cell apoptosis via inhibiting the PI3K-Akt-Bcl2 pathway.

Although arginine synthesis in adult swine is through the intestinal-renal axis, the neonatal intestine contains all the enzymes necessary to synthesize and transport arginine [20]. Arginine transport involves the system y+ (a high-affinity, Na+-independent transporter, CAT-1, CAT-2, CAT-3, CAT-4) and Na-dependent transporters (e.g., b^0,+^, B^0,+^, and y^+^L) in a cell-specific manner [21]. For intestinal epithelial cells, 70% Arginine is transported through the system y+. In this study, we found that increasing arginine and addition of L-NAME could both promote Arginine uptake. In order to research the regulatory mechanism, relative arginine transporters were determined. It reported that arginine uptake could been increased by activation of the inducible NOS (iNOS), associated with upregulation of CAT-2 in rats. And aortic arginine uptake is attenuated by inhibiting eNOS in hypercholesterolemic rats [22]. According to this present study, addition of L-NAME was found to increase the expressions of CAT-2 and y+LAT-1, suggesting that L-NAME might increase arginine influx via increasing the the expressions of CAT-2 and y+LAT-1.

To verify whether arginine directly increase the activation of mTOR signaling pathways and the interaction of arginine uptake and mTOR pathway, the relative protein was determined. Ban et al. and Rhoads et al. found that arginine could activate mTOR signaling pathways in intestinal epithelial cells [8, 23]. Tan et al. reported that Arginine could increase the expressions of phosphorylation of mTOR, S6K1 and 4EBP1 in IPEC-1 cells [3]. The present study shows that Arginine promotes the expressions of mTOR signaling pathways proteins, which is consistent with the previous study, and addition of L-NAME reduces the relative protein expressions of mTOR pathway. The previous study found that Arginine showed the relative increase (42.2%) in intracellular pools by inhibiting mTOR [24]. Visigalli et al. found that rapamycin would stimulate arginine influx through CAT2 transporters and mTOR activity might be associated with the repression of CAT2 expression at mRNA and protein level [25]. In our study, L-NAME could inhibit mTOR pathway, suggesting that L-NAME might promote the expressions of y+CAT-2 by inhibiting mTOR pathway

In conclusion, these findings indicated that Arginine-NO pathway could regulate cell growth and proliferation by inhibiting mTOR pathway and promoting PI3K-Akt-Bcl2 pathway and increase arginine influx via increasing the expressions of CAT-2 and y+LAT-1. These effects depended on the concentration of aginine supplementation. These results provide effective theoretical basis of Arginine application in piglets nutrition and also provide reference for using Arginine to remedy human intestinal disease.

## MATERIALS AND METHODS

### Reagents and cell culture

Cell culture was down according to a previous study [3]. Dulbecco's modified Eagle's F12 Ham medium (DMEM-F12), fetal bovine serum (FBS), and antibiotics were procured from Invitrogen (Grand Island, NY, USA). Epidermal growth factor was obtained from BD Biosciences (Bedford, MA, USA), and plastic culture plates were manufactured by Corning Inc. (Corning, NY, USA). Unless indicated, all other chemicals were purchased from Sigma-Aldrich (St. Louis, MO, USA). Briefly, IPEC-1 cells (passage 5–10) were grown in serial passage in uncoated plastic culture flasks (100 mm^2^) in DMEM-F12 containing 17.5 mM D-glucose, 2 mM Gln, 0.7 mM Arginine, 15 mM HEPES (pH 7.4), 5.0 % FBS, insulin (5.0 μg/ml), transferrin (5.0 μg/ml), selenium (5.0 ng/ml), epidermal growth factor (5.0 μg/L), penicillin (50 μg/ml), streptomycin (4.0 μg/ml), and 0.25 μg/ml amphotericin B (fungizone®). Medium was changed every 2 days. At confluence, cells were trypsinized and seeded in 6-well cell culture plates with approximately 30 × 10^4^ cells per well and maintained at 37°C in a 5% CO_2_ incubator. After an overnight incubation, the cells were starved for 6 hrs in Arginine-free DMEM. The cells were cultured in medium containing 100 μM Arginine, 350 μM Arginine, 100 μM Arginine+400 μM L-NAME, 350 μM Arginine+400 μM L-NAME The medium was changed every 2 days. The cells were collected for further research after 4 days’ incubation.

### Nitric ode measurement

We used Nitric Oxide Colorimetric Assay Kit to measure NO. And this kit provides an accurate, convenient measure of total nitrate/nitrite in a simple two-step process. The steps of measurement were performed following manufacturer's instructions for Nitric Oxide Colorimetric Assay Kit (BioVision, USA).

### EdU(5-Ethynyl -2′- deoxy uridine) measurement

IPEC-1 cells grown on 96-well plates after 96 hours incubation were labeled with 50 μM 5-ethynyl-2′-deoxyuridine (EdU; Invitrogen) for 30 minutes (pulse) before replacing with fresh medium. Cell fixation, permeabilization and EdU detection were performed following manufacturer's instructions for EdU kit (Invitrogen). Cells were measured using an inverted fluorescence microscope (DMI3000B, Leica, Germany)

### Flow cytometry

The cells were handled and fixed for flow cytometry essentially as described earlier [26]. Briefly, about 1 × 10^6^ cells were pelleted at 16 000 × g for 5 min. The supernatant was removed and 1 ml of 70% cold ethanol was slowly added during vigorous mixing. Samples were stored at 4°C. Samples were washed with PBS and resuspended in PBS containing 150 μg/ml RNase A. DNA was stained with 50 μg/ml propidium iodide for 1 h at room temperature. DNA content was then analyzed by FACS analysis on a Becton Dickinson FACSCanto.

### Arginine transport measurement

After a 4-day period of culture, the medium was removed and the cells were rinsed three times with “transport buffer” (37°C) comprised of 137 mmol/L choline Cl, 10 mmol/L HEPES/Tris buffer (pH 7.4), 4.7 mmol/L KCl, 1.2 mmol/L MgSO_4_, 1.2 mmol/L KH_2_PO_4_, 2.5 mmol/L CaCl_2_. Transport was initiated by simultaneously adding 1 ml of this buffer also containing L-[^3^H] arginineinine (2 μCi/ml, 5 μmol/L) into each transport well (24 wells/plate). Cell culture plates were continuously shaken by an orbital shaker (1 Hz) during the 5 minutes uptake period. Uptake was arrested by discarding the transport buffer and washing cells three times with ice-cold transport buffer lacking ^3^H-labeled substrate. The ^3^H radioactivity, extracted from the cells with 0.5 ml 1N NaOH and neutralized with 0.25 ml of acetic acid, was assayed by liquid scintillation spectrometry. Protein in the NaOH extract was measured using the BCA method. Arginine uptake rates are expressed as nmoles of Arginine per minute per milligram of cell protein.

### Real-time PCR

The protocol of total RNA extraction, quantification, cDNA synthesis and real-time PCR was adapted from the method of Li et al. [27 and 29]. Briefly, total RNA was isolated from cell samples by using the Trizol method. Real time PCR was carried out by using forward and reverse primers (Table 1) to amplify the target genes. For quantification, amplification efficiencies curves were constructed from serial 1:2 dilutions, and the 2^−ΔΔCT^ (ΔΔCT ={Ct(target)-Ct(relevant 18s or β-actin)}- ΔCt (average of control group)) method was used to calculate the mRNA expression of the target genes relative to housekeeping gene (β-actin or 18S).

**Table 1:** Forward and reverse primers of β-actin, y^+^CAT-1, y^+^CAT-2, and y^+^LAT-1

Target Gene	Primer Sequence (5′ to 3′)	Accession No.
β-actin	F: GGACCTGACCGACTACCTCA	XM_003124280.3
	R: CACAGCTTCTCCTTGATGTCC	
y^+^CAT-1	F: TCTGGTCCTGGGCTTCATAA	NM_001012613.1
	R: ACCTTCGTGGCATTGTTCAG	
y^+^CAT-2	F: ACAACTGGCGAAGAAGTCCG	NM_001110420.1
	R: CTGCCGAGACCCCAAAATAG	
y^+^LAT-1	F: GAGTGCCAGAACACAAACGA	NM_001110421.1
	R: TCCTCCATCTTCCAAATCCA	

### Western blotting analysis

Frozen cell samples were collected as described by Tan et al. [14]. Protein concentrations of cell homogenates were measured by using the BCA method and bovine serum albumin as standard. All samples were adjusted to an equal concentration (50 μg protein). The western blotting was conducted based on previous description [28]. The primary antibodies are 4EBP1 (1:1,000; Cell Signaling Technology), phosphorylated 4EBP1(Ser65) (1:1,000; Cell Signaling Technology), mTOR (1:1,000; Cell Signaling Technology), phosphorylated mTOR (Ser2448) (1:1,000; Cell Signaling Technology), phosphorylated p70S6 kinase (Thr389) (1:1,000; Cell Signaling Technology), CAT-1(1:400; Santa Cruz Biotechnology, Dallas, TX), CAT-2(1:400; Santa Cruz Biotechnology, Dallas, TX), phosphorylated phosphatidylinositol 3-kinase (1:1,000; Cell Signaling Technology), Akt (Protein Kinase B; 1:1,000; Cell Signaling Technology), phosphorylated Akt [p-Akt (Ser 473); 1:1,000; Cell Signaling Technology), Bcl2 (1:1000; LifeSpan BioSciences, Inc), or β-actin (1:400; Santa Cruz Biotechnology, Dallas, TX). All protein measurements were normalized to β-actin.

### Statistical analysis

Results are expressed as Mean±SEM. The statistical analysis was performed by one-way ANOVA using SPSS 17.0 (SPSS Inc., Chicago, IL, USA). Probability values < 0.05 were considered statistically significant.

## Abbreviations

NO: Nitric oxide
IPEC-1: Intestinal porcine epithelial cells
DMEM-F12: Dulbecco's modified Eagle’s-F12 Ham medium
NOS: Nitric oxide synthase
mTOR: Mammalian target of rapamycin
PI3K: Phosphatidyl Inositol 3-kinase
L-NAME: L-nitro-arginine methylester
p70 S6: p70 ribosomal protein S6 kinase
4E-BP1: Eukaryotic initiation factor 4E-binding protein 1
eNOS: Endothelial nitric oxide synthase
EdU: 5-ethynyl-2′-deoxyuridine.
